# Supplementary material for: Climate, soil or both? Which variables are better predictors of the distributions of Australian shrub species?
Source: PeerJ. 2017 Jun 22;5:e3446. doi: 10.7717/peerj.3446 (PMC5483041; doi:10.7717/peerj.3446)
Supplement: Table S1 — Correlation coefficients for nine environmental variables used as predictors to fit the species distribution models. Mean Annual Temperature (T), Maximum Temperature of the Warmest Month (TMwarm), Mean Annual Precipitation (P), Precipitation of the Warmest Quarter (PQwarm), Precipitation of the Coldest Quarter (PQcold), bulk density (BD), clay content percentage (CLAY), pH CaCl2 (pH), and organic carbon (OC). [file peerj-05-3446-s001.docx]

**Table S1: Correlation matrix of nine environmental variables used in models.**

Correlation coefficients for nine environmental variables used as predictors to fit the species distribution models. Mean Annual Temperature (T), Maximum Temperature of the Warmest Month (TMwarm), Mean Annual Precipitation (P), Precipitation of the Warmest Quarter (PQwarm), Precipitation of the Coldest Quarter (PQcold), bulk density (BD), clay content percentage (CLAY), pH CaCl_2_ (pH), and organic carbon (OC).

|  | T | TMwarm | P | PQwarm | PQcold | BD | CLAY | pH | OC |
| --- | --- | --- | --- | --- | --- | --- | --- | --- | --- |
| T | 1.00 | 0.79 | 0.12 | 0.46 | -0.73 | 0.49 | -0.07 | -0.17 | -0.58 |
| TMwarm | 0.79 | 1.00 | -0.40 | -0.03 | -0.77 | 0.65 | -0.09 | 0.05 | -0.74 |
| P | 0.12 | -0.40 | 1.00 | 0.83 | 0.31 | -0.49 | 0.10 | -0.43 | 0.49 |
| PQwarm | 0.46 | -0.03 | 0.83 | 1.00 | -0.11 | -0.23 | 0.10 | -0.40 | 0.15 |
| PQcold | -0.73 | -0.77 | 0.31 | -0.11 | 1.00 | -0.60 | 0.13 | -0.12 | 0.76 |
| BD | 0.49 | 0.65 | -0.49 | -0.23 | -0.60 | 1.00 | -0.24 | 0.18 | -0.79 |
| CLAY | -0.07 | -0.09 | 0.10 | 0.10 | 0.13 | -0.24 | 1.00 | 0.48 | 0.10 |
| pH | -0.17 | 0.05 | -0.43 | -0.40 | -0.12 | 0.18 | 0.48 | 1.00 | -0.33 |
| OC | -0.58 | -0.74 | 0.49 | 0.15 | 0.76 | -0.79 | 0.10 | -0.33 | 1.00 |
