# Supplementary material for: Climate, soil or both? Which variables are better predictors of the distributions of Australian shrub species?
Source: PeerJ. 2017 Jun 22;5:e3446. doi: 10.7717/peerj.3446 (PMC5483041; doi:10.7717/peerj.3446)
Supplement: Data S1 — Links for datasets used in the research. [file peerj-05-3446-s003.docx]

Climate data:

<http://wallaceinitiative.org/climate_2012/tdhtools/Search/DataDownload.php>

Soil data:

<http://www.clw.csiro.au/aclep/soilandlandscapegrid/index.html>

<http://www.clw.csiro.au/aclep/soilandlandscapegrid/GetData-GIS.html>

**Table 1: Shrub species used in fitting models and their source data.**

29 shrub species names and families and the final number of each species used in species distribution models after removing unreliable species data. Final column shows source data link from the Atlas of Living Australia (ALA, <http://www.ala.org.au/>) from which species occurrence records were downloaded.

| **Family** | **Species** | **Final record available for modelling** | **Data link** |
| --- | --- | --- | --- |
| Asteraceae | *Ozothamnus turbinatus* | 54 | [*http://biocache.ala.org.au/occurrences/search?q=lsid:http://id.biodiversity.org.au/node/apni/2898660#tab_recordsView*](http://biocache.ala.org.au/occurrences/search?q=lsid:http://id.biodiversity.org.au/node/apni/2898660#tab_recordsView) |
| Casuarinaceae | *Allocasuarina campestris* | 407 | [*http://biocache.ala.org.au/occurrences/search?q=lsid:http://id.biodiversity.org.au/node/apni/2893713#tab_recordsView*](http://biocache.ala.org.au/occurrences/search?q=lsid:http://id.biodiversity.org.au/node/apni/2893713#tab_recordsView) |
| Chenopodiaceae | *Atriplex angulata* | 516 | [*http://biocache.ala.org.au/occurrences/search?q=lsid:http://id.biodiversity.org.au/node/apni/2887187*](http://biocache.ala.org.au/occurrences/search?q=lsid:http://id.biodiversity.org.au/node/apni/2887187) |
|  | *Atriplex eardleyae* | 481 | [*http://biocache.ala.org.au/occurrences/search?q=lsid:http://id.biodiversity.org.au/node/apni/2895815*](http://biocache.ala.org.au/occurrences/search?q=lsid:http://id.biodiversity.org.au/node/apni/2895815) |
|  | *Atriplex holocarpa* | 1025 | [*http://biocache.ala.org.au/occurrences/search?q=lsid:http://id.biodiversity.org.au/node/apni/2919449*](http://biocache.ala.org.au/occurrences/search?q=lsid:http://id.biodiversity.org.au/node/apni/2919449) |
|  | *Atriplex nummularia* | 829 | [*http://biocache.ala.org.au/occurrences/search?q=lsid:http://id.biodiversity.org.au/node/apni/2890564*](http://biocache.ala.org.au/occurrences/search?q=lsid:http://id.biodiversity.org.au/node/apni/2890564) |
|  | *Atriplex vesicaria* | 2931 | [*http://biocache.ala.org.au/occurrences/search?q=lsid:http://id.biodiversity.org.au/node/apni/2901256*](http://biocache.ala.org.au/occurrences/search?q=lsid:http://id.biodiversity.org.au/node/apni/2901256) |
|  | *Maireana aphylla* | 1215 | [*http://biocache.ala.org.au/occurrences/search?q=lsid:http://id.biodiversity.org.au/node/apni/2902869*](http://biocache.ala.org.au/occurrences/search?q=lsid:http://id.biodiversity.org.au/node/apni/2902869) |
| Myrtaceae | *Epacris impressa* | 1523 | [*http://biocache.ala.org.au/occurrences/search?q=lsid:http://id.biodiversity.org.au/node/apni/2894437*](http://biocache.ala.org.au/occurrences/search?q=lsid:http://id.biodiversity.org.au/node/apni/2894437) |
|  | *Acacia aneura* | 3290 | [*http://biocache.ala.org.au/occurrences/search?q=lsid:http://id.biodiversity.org.au/node/apni/2912469*](http://biocache.ala.org.au/occurrences/search?q=lsid:http://id.biodiversity.org.au/node/apni/2912469) |
|  | *Acacia sclerosperma* | 314 | [*http://biocache.ala.org.au/occurrences/search?q=lsid:http://id.biodiversity.org.au/node/apni/2901555*](http://biocache.ala.org.au/occurrences/search?q=lsid:http://id.biodiversity.org.au/node/apni/2901555) |
|  | *Acacia tetragonophylla* | 463 | [*http://biocache.ala.org.au/occurrences/search?q=lsid:http://id.biodiversity.org.au/node/apni/2896531*](http://biocache.ala.org.au/occurrences/search?q=lsid:http://id.biodiversity.org.au/node/apni/2896531) |
|  | *Acacia victoriae* | 1712 | [*http://biocache.ala.org.au/occurrences/search?q=lsid:http://id.biodiversity.org.au/node/apni/2904053*](http://biocache.ala.org.au/occurrences/search?q=lsid:http://id.biodiversity.org.au/node/apni/2904053) |
|  | *Eucalyptus diversifolia* | 716 | [*http://biocache.ala.org.au/occurrences/search?q=lsid:http://id.biodiversity.org.au/node/apni/2894183*](http://biocache.ala.org.au/occurrences/search?q=lsid:http://id.biodiversity.org.au/node/apni/2894183) |
|  | *Eucalyptus dumosa* | 1759 | [*http://biocache.ala.org.au/occurrences/search?q=lsid:http://id.biodiversity.org.au/node/apni/2894653*](http://biocache.ala.org.au/occurrences/search?q=lsid:http://id.biodiversity.org.au/node/apni/2894653) |
|  | *Eucalyptus gracilis* | 1998 | [*http://biocache.ala.org.au/occurrences/search?q=lsid:http://id.biodiversity.org.au/node/apni/2905257*](http://biocache.ala.org.au/occurrences/search?q=lsid:http://id.biodiversity.org.au/node/apni/2905257) |
|  | *Eucalyptus incrassata* | 1640 | [*http://biocache.ala.org.au/occurrences/search?q=lsid:http://id.biodiversity.org.au/node/apni/2887758*](http://biocache.ala.org.au/occurrences/search?q=lsid:http://id.biodiversity.org.au/node/apni/2887758) |
|  | *Eucalyptus oleosa* | 1924 | [*http://biocache.ala.org.au/occurrences/search?q=lsid:http://id.biodiversity.org.au/node/apni/2921021*](http://biocache.ala.org.au/occurrences/search?q=lsid:http://id.biodiversity.org.au/node/apni/2921021) |
|  | *Eucalyptus socialis* | 2817 | [*http://biocache.ala.org.au/occurrences/search?q=lsid:http://id.biodiversity.org.au/node/apni/2914882*](http://biocache.ala.org.au/occurrences/search?q=lsid:http://id.biodiversity.org.au/node/apni/2914882) |
|  | *Leptospermum continentale* | 798 | [*http://biocache.ala.org.au/occurrences/search?q=lsid:http://id.biodiversity.org.au/node/apni/2917147*](http://biocache.ala.org.au/occurrences/search?q=lsid:http://id.biodiversity.org.au/node/apni/2917147) |
|  | *Leptospermum glaucescens* | 225 | [*http://biocache.ala.org.au/occurrences/search?q=lsid:http://id.biodiversity.org.au/node/apni/2913452*](http://biocache.ala.org.au/occurrences/search?q=lsid:http://id.biodiversity.org.au/node/apni/2913452) |
|  | *Leptospermum laevigatum* | 159 | [*http://biocache.ala.org.au/occurrences/search?q=lsid:http://id.biodiversity.org.au/node/apni/2905839*](http://biocache.ala.org.au/occurrences/search?q=lsid:http://id.biodiversity.org.au/node/apni/2905839) |
|  | *Leptospermum lanigerum* | 1269 | [*http://biocache.ala.org.au/occurrences/search?q=lsid:http://id.biodiversity.org.au/node/apni/2910654*](http://biocache.ala.org.au/occurrences/search?q=lsid:http://id.biodiversity.org.au/node/apni/2910654) |
|  | *Leptospermum scoparium* | 979 | [*http://biocache.ala.org.au/occurrences/search?q=lsid:http://id.biodiversity.org.au/node/apni/2896906*](http://biocache.ala.org.au/occurrences/search?q=lsid:http://id.biodiversity.org.au/node/apni/2896906) |
|  | *Melaleuca ericifolia* | 395 | [*http://biocache.ala.org.au/occurrences/search?q=lsid:http://id.biodiversity.org.au/node/apni/2891096*](http://biocache.ala.org.au/occurrences/search?q=lsid:http://id.biodiversity.org.au/node/apni/2891096) |
|  | *Melaleuca squamea* | 483 | [*http://biocache.ala.org.au/occurrences/search?q=lsid:http://id.biodiversity.org.au/node/apni/2893344*](http://biocache.ala.org.au/occurrences/search?q=lsid:http://id.biodiversity.org.au/node/apni/2893344) |
|  | *Melaleuca squarrosa* | 678 | [*http://biocache.ala.org.au/occurrences/search?q=lsid:http://id.biodiversity.org.au/node/apni/2897568*](http://biocache.ala.org.au/occurrences/search?q=lsid:http://id.biodiversity.org.au/node/apni/2897568) |
| Sapindaceae | *Atalaya hemiglauca* | 1771 | [*http://biocache.ala.org.au/occurrences/search?q=lsid:http://id.biodiversity.org.au/node/apni/2908904*](http://biocache.ala.org.au/occurrences/search?q=lsid:http://id.biodiversity.org.au/node/apni/2908904) |
| Scrophulariaceae | *Eremophila freelingii* | 1044 | [*http://biocache.ala.org.au/occurrences/search?q=lsid:http://id.biodiversity.org.au/name/apni/241348*](http://biocache.ala.org.au/occurrences/search?q=lsid:http://id.biodiversity.org.au/name/apni/241348) |
